# The health impact of trade and investment agreements: a quantitative systematic review and network co-citation analysis

**DOI:** 10.1186/s12992-017-0240-x

**Published:** 2017-03-08

**Authors:** Pepita Barlow, Martin McKee, Sanjay Basu, David Stuckler

**Affiliations:** 1 Department of Sociology, University of Oxford, Manor Road Building, Manor Road, OX1 3UQ Oxford, UK; 2 Department of Public Health and Policy, London School of Hygiene & Tropical Medicine, London, UK; 3 Stanford Prevention Research Center, Stanford University, Stanford, USA

**Keywords:** Trade and investment agreements (RTAs), Trade policy, Foreign investment policy, Diets, Tobacco, Non-communicable diseases, Health outcomes, Systematic review, Co-citation analysis

## Abstract

**Background:**

Regional trade agreements are major international policy instruments that shape macro-economic and political systems. There is widespread debate as to whether and how these agreements pose risks to public health. Here we perform a comprehensive systematic review of quantitative studies of the health impact of trade and investment agreements. We identified studies from searches in PubMed, Web of Science, EMBASE, and Global Health Online. Research articles were eligible for inclusion if they were quantitative studies of the health impacts of trade and investment agreements or policy. We systematically reviewed study findings, evaluated quality using the Quality Assessment Tool from the Effective Public Health Practice Project, and performed network citation analysis to study disciplinary siloes.

**Results:**

Seventeen quantitative studies met our inclusion criteria. There was consistent evidence that implementing trade agreements was associated with increased consumption of processed foods and sugar-sweetened beverages. Granting import licenses for patented drugs was associated with increased access to pharmaceuticals. Implementing trade agreements and associated policies was also correlated with higher cardiovascular disease incidence and higher Body Mass Index (BMI), whilst correlations with tobacco consumption, under-five mortality, maternal mortality, and life expectancy were inconclusive. Overall, the quality of studies is weak or moderately weak, and co-citation analysis revealed a relative isolation of public health from economics.

**Conclusion:**

We identified limitations in existing studies which preclude definitive conclusions of the health impacts of regional trade and investment agreements. Few address unobserved confounding, and many possible consequences and mechanisms linking trade and investment agreements to health remain poorly understood. Results from our co-citation analysis suggest scope for greater interdisciplinary collaboration. Notwithstanding these limitations, our results find evidence that trade agreements pose some significant health risks. Health protections in trade and investment treaties may mitigate these impacts.

**Electronic supplementary material:**

The online version of this article (doi:10.1186/s12992-017-0240-x) contains supplementary material, which is available to authorized users.

## Background

**Fig. 1:**
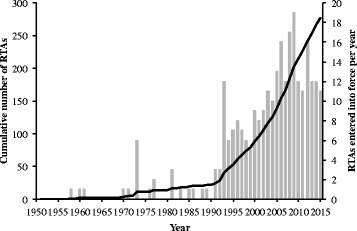
Number of RTAs, 1950–2010. *Notes*: Solid black line represents cumulative number of RTAs in force; grey bars show RTAs entered into force per year. Data extracted from the World Trade Organization’s Regional Trade Agreements Information System on 15 February 2016. See bibliography for full reference [57]

Regional trade and investment agreements (RTAs) are increasingly being used to promote international integration and economic growth. This has taken particular prominence as world trade has slowed in the wake of the 2008 global financial crisis: in 1990 there were 22 bi-lateral and regional RTAs, which rose to over 270 by 2016, as shown in Fig. 1 [1, 2]. Two large RTAs have been at the centre of recent negotiations: the Trans-Pacific Partnership, between the US and 11 Pacific Rim countries, signed in February 2016; and the Transatlantic Trade and Investment Partnership, between the US and the European Union (EU), signed in October 2016 [3]. These RTAs are as much about facilitating trade as reducing barriers to investment, such as by creating investor protections and by enabling private companies to participate in public sector procurement [4].

There has long been debate about the overall impact of RTAs on populations, not just economically, but also in terms of health and well-being [5–7]. In 2015, disputes about health in the World Trade Organization’s (WTO) Technical Barriers to Trade Committee reached record numbers. The WTO notes that protecting health is a dominant trade concern among members who seek to “strike a balance between trade and health” as they face potential economic costs and legal challenges when introducing new health measures [8, 9].

Concerned public health advocates argue that RTAs pose health risks through a number of mechanisms. One is that they could worsen dietary quality through enhancing transnational trade in unhealthy foods enriched with salt, sugar and fat [10–12]. Another is that RTAs could facilitate the spread of tobacco and alcohol use by weakening public health programmes, as well as increase the prices of pharmaceuticals by extending patent protections [13, 14]. In contrast, advocates of RTAs point to several benefits that achieve progress towards the Sustainable Development Goals [15]. This includes increasing the security of food supply systems and reducing malnutrition, as well as improved access to pharmaceuticals, either through importation or foreign investment [16–18].

**Fig. 2:**
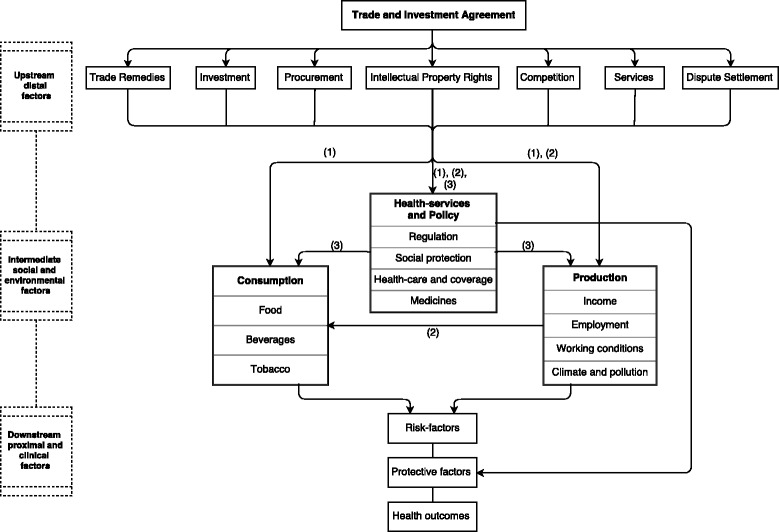
Macro-social model outlining potential health effects of RTAs and associated mechanisms. *Notes*: Numbers 1–3 identify whether outcomes and pathways are linked indirectly via changes to (1) trade flows, (2) foreign direct investment flows (FDI), or (3) directly via RTA clauses. Relevant RTA provisions were identified from Dur and Baccini (2014)

Previous studies of RTAs have been published in disparate disciplines and have analysed extensively the impact of RTAs on a range of political and economic phenomena [4]. Studies of the health impacts of RTAs, together with their broader social consequences, are conspicuous by their absence from this empirical literature. A number of possible mechanisms linking RTAs to health are shown in the conceptual framework in Fig. 2, which incorporates findings from different disciplines, including social epidemiology, public health, political economy and economics. The conceptual framework treats RTAs as a distal upstream determinant of population health and health equity via their impact on social and environmental factors that influence downstream proximal determinants of health outcomes [19–21]. The framework focuses on three main intermediate mechanisms linking RTAs to health outcomes: production, consumption, and health-services and policy. This builds on previous identification by Labonte and Schrecker (2007), Blouin et al. (2009), and Friel et al. (2015) [13, 22, 23].

First, RTAs can impact consumption via increased importation of food, soft-drinks and alcoholic beverages, and tobacco products. This increased competition from international imports can also lead domestic firms to lower prices, so further increasing consumption. Second, RTAs can alter the scope and scale of production through trade, Foreign Direct Investment (FDI) and subsequent competition with domestic firms. This can impact incomes, including levels and degree of inequality, employment, job security, and working conditions, and may have additional consequences for the environment via pollution and climate change. Changes to production also affect consumption of food, beverages and tobacco products via increased foreign investment in domestic production and intensified local competition, or via changing incomes and demand.

Third, RTAs have specific clauses which may impact health-services and policy. These can, for example, establish public procurement rules, investor protections and dispute settlement procedures that impact the ability or willingness of governments to introduce new regulations or policies that protect health, such as food and tobacco labelling (so-called ‘regulatory chill’). The nature of such regulations or, indeed, their absence, can, in turn, also impact the scope and scale of production and consumption. As a result, RTAs can have a substantial effect on health and access to care via changes to the availability of medicines, social protection, and health-service coverage: ‘protective factors’ that determine the extent to which risk-factor exposure ultimately impacts peoples’ health.

RTAs may therefore impact population health and health equity, for better or for worse, via myriad and complex pathways. Yet, a comprehensive understanding of these mechanisms and consequences is currently limited as previous systematic reviews did not scrutinise major pathways to impact. These include, among others, alcohol, tobacco, public health policymaking, and social protection programmes. Instead, previous reviews were relatively narrow in scope, with one previous review of the effects of trade and FDI on health systems, a second on non-nutritive health outcomes, and a third on the effects of RTAs on food environments [24–26]. Other analyses of RTAs and health are summaries, theoretical overviews, and policy commentaries, with contradictory claims [7, 12, 14, 27, 28]. The quality of research also varies considerably, as research designs span case study approaches to rigorous quasi-experimental methodologies, but most previous reviews and commentaries did not evaluate study quality [29, 30]. Here we address these gaps in the literature by reviewing high-quality, quantitative studies of the impact of RTAs on a wide range of health outcomes and corresponding pathways.

## Methods

### Selection criteria

**Fig. 3:**
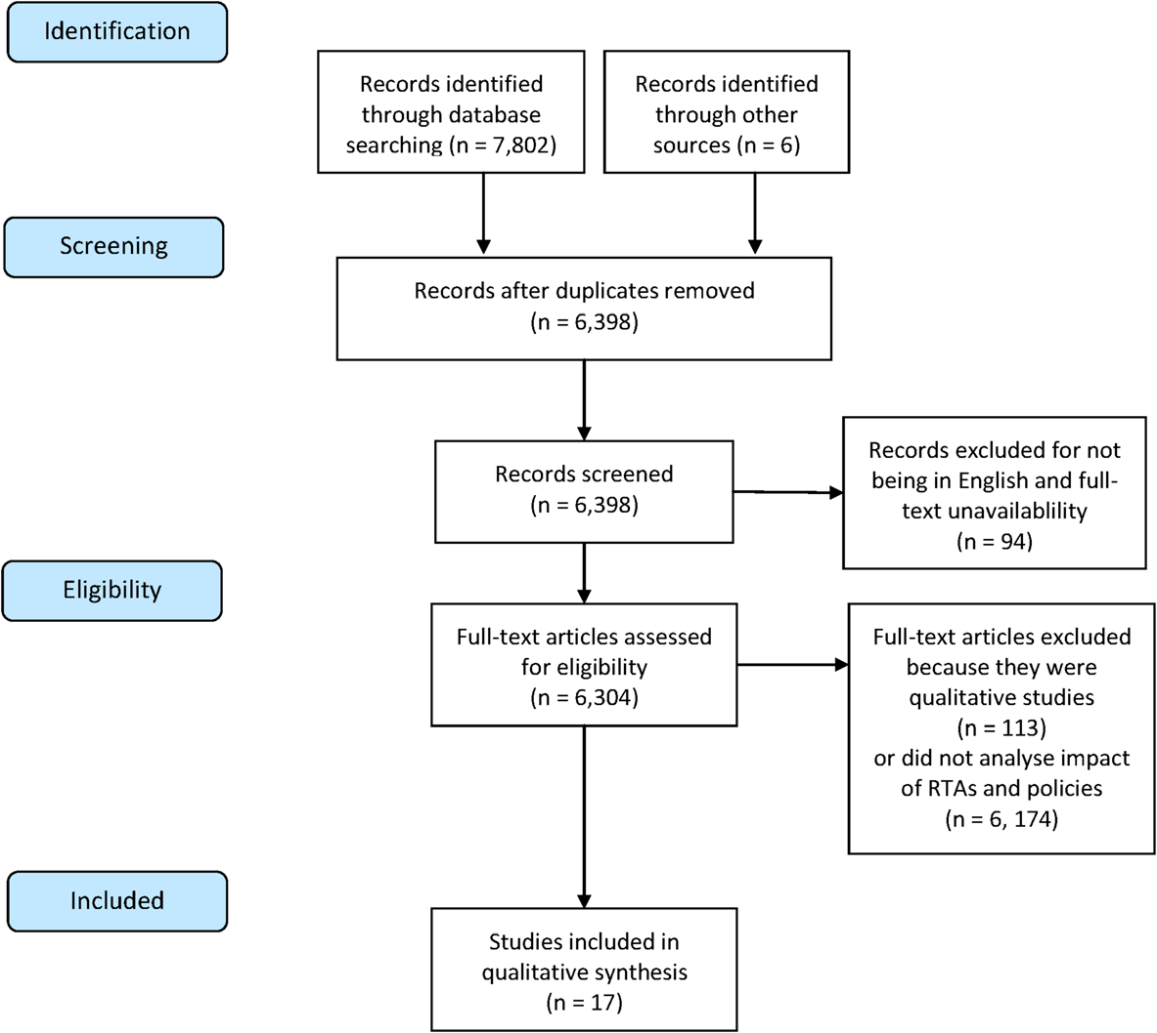
PRISMA diagram showing study identification, screening, and inclusion. *Notes*: flow diagram based on PRISMA guidelines set out in Moher et al. 2009

Figure 3 shows the PRISMA diagram depicting our study identification, screening and exclusion procedures. First, we searched Web of Science, Scopus, PubMed, EMBASE, and Global Health Online on 19 January 2016 for articles published since 1960. Following the methodology of Friel and colleagues (2013) we used terms that captured studies of trade and investment policies that were not analysed in the context of a specific RTA but are common components of many RTAs. Additional file 1: describes our search operators in detail. This search identified 7,802 papers. To ensure completeness we reviewed the bibliographies of commentaries and book chapters and searched the websites of relevant international organisations including the World Health Organisation and WTO, identifying an additional 6 studies.

Out of 7,808 identified studies we excluded duplicates (*n* = 1,410 studies), non-English studies (*n* = 90 studies), those which were not full articles (*n* = 4 studies), analyses which did not study the health effects RTAs or trade policies (*n* = 6,174 studies), and qualitative case studies (*n* = 113 studies). To review study findings we then extracted details of the study’s title, author, countries, years, research question, study design, measurement, method of analysis, and main findings. We then categorised study findings according to the macro-social framework, grouping studies into three categories: i) food, beverage and tobacco consumption, ii) access to medicines, and iii) overall health outcomes. We conducted our systematic review according to the PRISMA guidelines set out in Moher et al. (2009) (see Additional file 1: for full PRISMA checklist).

### Quality assessment

To rate methodological quality and evaluate risk of bias we used an updated version of the Quality Assessment Tool from the Effective Public Health Practice Project [31]. This scores studies between 1 and 3 in six components, measuring the strength of: (1) study design, (2) selection bias, (3) confounders, (4) data collection, (5) data analysis, and (6) reporting (see Additional file 1) [32].

### Network co-citation analysis

Since the prospective links between RTAs and health integrate findings from disparate disciplines we analyse co-citation patterns to identify cross-disciplinary fertilisation of included studies. Co-citation measures the frequency with which two documents are cited together by other documents. We analyse co-citation patterns to assess whether insights from different fields are being acknowledged with one another or are instead located in disciplinary siloes. As has been noted elsewhere, this is important as a failure to include work from other disciplines could lead to partial or incorrect conclusions [33–35]. To map co-citation patterns we extracted citation data from PubMed and Web of Science and analysed citation patterns using network-clustering algorithms in VOSviewer 1.6.1 [36].

### Funder involvement

The funder of the study had no role in study design, data collection, data analysis, data interpretation, or writing of the report. The corresponding author had full access to all the data in the study and had final responsibility for the decision to submit for publication.

## Results

### Study characteristics

Searches and exclusion identified 17 quantitative research articles which are summarised in Table 1. Eleven studies analysed the impact of RTAs on changes in the availability and consumption of food, tobacco and beverages. One study analysed the associations between RTAs and access to pharmaceuticals. Six studies analysed associations of RTAs and trade policies with health outcomes including rates of under-five and maternal mortality, life expectancy, Body Mass Index (BMI), and cardiovascular disease incidence.

**Table 1:** Studies of RTAs and health meeting inclusion criteria

Source	Countries studied	Years studied	Analysis	Category
Bozorgmehr and San Sebastian 2014 [47]	22 high TB-burden countries	1990–2010	Multivariate	Health outcomes (TB and HIV incidence)
Chaloupka and Laixuthai 1996 [43]	10 Asian countries	1970–1991	Multivariate	Cigarettes
Chatterjee et al., 2011 [37]	India	1990–2006	Bivariate	Food
Vogli RD et al. 2014 [49]	127 low-, middle- and high-income countries	1980–2008	Multivariate	BMI
Drope and Chavez, 2014 [42]	9 Southeast Asian countries	1999–2012	Bivariate	Cigarettes
Goryakin et al., 2015 [48]	56 low- and middle-income countries	1991–2009	Multivariate	BMI
Hawkes 2007	Central America (Honduras, El Salvador, Nicarague, Costa Rica, Guatemala), India, South Africa, Bangladesh, Uganda	various	Bivariate	Food
Hawkes 2010 [12]	Brazil, Argentina, Indonesia, Malaysia, China and India	1990–2005	Bivariate	Food
Schram et al. 2013 [39]	48 Sub-Saharan African countries	1995–2012	Bivariate	Food and beverages
Schram et al. 2015 [29]	Vietnam and The Philippines	1999–2013	Natural experiment	Beverages
Sharif et al. 2008 [40]	Pakistan	1993–2005	Multivariate	Health outcomes (mortality)
Stuckler et al. 2012 [41]	80 low- and middle- income countries	1997–2010	Multivariate	Food and beverages
Tausch 2015 [46]	99 low-, middle- and high-income countries	1970–2005	Multivariate	Health outcomes (mortality)
Thow and Hawkes 2009 [30]	Honduras, Costa Rica, Guatemala, El Salvador, Nicaragua	1990–2006	Bivariate	Food
Thow and Snowdon 2010 [39]	10 Pacific Island countries	1961–2005	Bivariate	Food
Umana-Pena et al. 2014 [45]	WTO member countries	1995–2010	Multivariate	Health outcomes (mortality)
Yamabhai et al. 2011 [41]	Thailand	2006–2013	Bivariate	Medicines and medical technologies

### i) Impact of RTAs on consumption: food, beverages and tobacco

Among the eleven studies in this category, six were bivariate analyses, four were multivariate analyses, and one used a natural experiment design. Overall, entering into trade agreements and implementing associated policies were correlated with increases in imports and consumption of edible oils, meats, processed foods, and sugar-sweetened beverages. Studies of tobacco consumption reported contrasting results.

### Food

The five bivariate analyses identified increased imports, supply, sales and consumption of processed foods, edible oils and meats after reducing tariffs on these products. For example, Chatterjee et al. identify an 111% increase in edible oil imports, a decrease in consumption of cereal items, and a 12.5% rise in processed food consumption in urban areas after tariffs were reduced in India after liberalisation reforms in the 1990s [37]. A further four studies identified increases in the supply of animal products, processed foods and staple grains after tariffs fell in Central America, Latin America, Africa, the Pacific, and Southeast Asia [12, 30, 38].

Two multivariate studies analysed the associations between trade agreements and the demand for staple grains and processed foods. Schram et al. use structural equation modelling to analyse the association between the KOF (Konjunkturforschungsstelle Swiss Economic Institute) economic globalisation index and per capita sales of multiple food products in 48 Sub-Saharan African countries 1995–2012. The authors’ identify an increase in foreign investment inflows, imports of high-sugar and energy-dense foods, grocery retail sales, and daily caloric intake in all countries [39]. Sharif et al. (2008) analyse the effect of the 1994 Uruguay round of trade liberalisation agreements on changes to the production and consumption of wheat and rice in Pakistan, 1993–2005, and identify a rise in the wholesale price of rice and wheat production and a corresponding reduction in demand [40].

### Beverages

One multivariate study by Stuckler et al. analysed the association between entering into RTAs with the United States and soft-drinks consumption in 50 low- and middle-income countries. They identify 63.4% higher levels of soft-drink consumption per capita in countries with US RTAs [41]. One study analysed the effects of RTAs on beverage consumption using quasi-experimental methods. Schram et al. (2015) analysed a natural experiment in Vietnam and the Philippines to evaluate the effect of joining the WTO upon the sales of sugar-sweetened beverages. The authors find that after Vietnam removed trade restrictions to join the WTO, the growth rate of soft-drink sales per capita rose 4.6 L per annum faster in Vietnam compared with the Philippines [29].

### Tobacco

One bivariate study by Drope et al. analysed the association between changes to cigarette tariffs and cigarette consumption per capita in Southeast Asia in 1999 and 2012 and reported no universal association [42]. A multivariate analysis by Chaloupka et al. estimated cross-national longitudinal fixed-effects models to study the association between legal disputes with the US tobacco industry and cigarette consumption in 10 Asian countries, 1970–1992. The authors estimate that removing tobacco industry protections in accordance with the resolution agreement increased per capita cigarette consumption by nearly 10% by 1991 [43].

### ii) Impact of RTAs on access to medicines and technologies

We identified one study which analysed associations between trade policy and access to pharmaceuticals. Yambhai et al. (2011) analysed the effects of granting import licenses for seven generic equivalents of domestically patented drugs in Thailand, 2002–2008. By calculating differences between extrapolated pre-intervention and observed post-intervention access rates the authors estimated that an additional 84,158 patients received access to the drugs and 12,493 QALYs were gained due to the reforms [44].

### iii) Impact of RTAs on overall health outcomes

Six studies analysed the effects of RTAs and trade policies on health outcomes, including rates of under-five and maternal mortality, life expectancy, tuberculosis incidence, Body Mass Index (BMI), and cardiovascular disease incidence. All six studies used multivariate statistical methods. Overall, implementing liberalisation policies and trade agreements was linked with higher BMIs and cardiovascular disease incidence, but there was no consistent association with under-five and maternal mortality, life expectancy and tuberculosis incidence.

Turning first to mortality and life expectancy, Umana-Pena et al. (2015) asked whether there was an association with liberalisation in the service sector in WTO member countries, 1994–2010. In cross-sectional regression models higher levels of service sector liberalisation were associated with lower maternal and infant mortality and longer life-expectancy, but greater progress in liberalisation between 1995 and 2010 was not associated with changes in under-5 mortality, maternal mortality, or life expectancy [45]. In contrast, Tausch et al. studied the association between the economic globalisation component of the KOF index and infant mortality in 99 low-, middle- and high-income countries, 1970–2005, finding that greater restrictions on FDI and trade were associated with higher rates of infant mortality [46].

Bozorgmehr et al. (2014) analysed the association of tuberculosis incidence with four liberalisation indicators: WTO membership, duration of membership, the trade and investment components of the KOF index, and the Freedom House Economic Freedom index, in 22 high-burden countries, 1990–2010. The authors find WTO member countries had significantly higher tuberculosis incidence rates compared with non-members. Results for the three other indicators were insignificant [47].

Turning to studies of BMI and cardiovascular disease, Goryakin et al. report a positive association between the probability of being overweight and a country’s degree of globalisation. The authors used multi-level longitudinal models combining country- and individual-level data for women in 56 low- and middle-income countries, 1992–2009. The authors report that the association between BMI and globalisation was stronger for social and political dimensions of the KOF index compared with the economic dimension [48]. De Vogli et al. estimate longitudinal fixed-effects models in a sample of 127 countries, 1980–2008 and also report a positive association between the economic globalization component of the KOF index and mean BMI [49]. Finally, the studies of processed food and beverage consumption by Stuckler et al. (2012) and Schram et al. (2013) also reported a positive association of implementing RTAs with rates of cardiovascular disease, overweight and obesity prevalence [39, 41].

### Network co-citation analysis

**Fig. 4:**
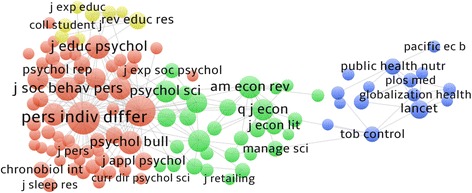
Co-citation of studies. *Notes:* Created using VOSViewer Version 1.6.1. The network map shows co-citation patterns of the 117 journals cited at least 5 times within the studies we reviewed. Node size corresponds to the number of citations, lines correspond to the existence of a citation in either direction, and distance between nodes corresponds to the tendency for studies to be cited together by other studies

Included studies were published most frequently in ‘Globalization and Health’ (3 studies) with the remainder spread across journals public health and social science (10 studies), book chapters (2 studies) and empirical policy reports (2 studies) (see Additional file 1). Figure 4 shows the results from our co-citation analysis.

Four clusters are visible in the psychology, social psychology, economics, and public health disciplines. There is a strong tendency for co-citation of studies in psychology and social psychology. Co-citations were weaker for public health and economics and weakest for public health and psychology.

### Methodological quality assessment

**Fig. 5:**
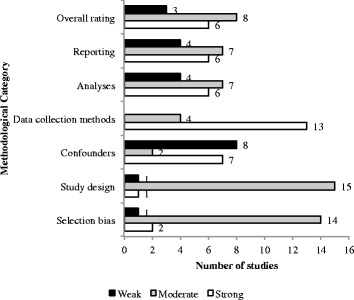
Methodological quality assessment: distribution of studies between quality scores. *Notes:* Studies reviewed = 17. Studies were evaluated using an adapted version of the Quality Assessment Tool developed by the Effective Public Health Practice Project (Thomas et al. [31]). See Additional file 1: for full details

Out of the seventeen studies included in our review, fifteen analysed repeated cross-sections of country-level data, one study analysed cross-sectional regional and firm-level data, and one study analysed repeated cross-sectional individual level data. Eight studies used bivariate time-series designs, eight used multivariate statistics, and one analysed a natural experiment. Figure 5 shows the results from the full quality assessment of included studies. Overall, six out of seventeen studies were rated as ‘strong’ on overall methodological quality and risk of bias; eight studies were rated as ‘moderate’ and three studies were rated ‘weak’.

Trade and investment measures varied in specificity. Five studies analysed product-specific changes in RTA policies such as the tariff rate, and five studies constructed dichotomous indicators of participating in RTAs. Three studies measured general changes to trade or investment policy, for example mean tariff rates. A further five studies used an index, four of which used the economic globalisation component of the KOF index which is a composite measure of changes to both restrictions and flows of trade and investment [50].

## Discussion

Five main findings can be drawn from our review. First, implementing RTAs and related policies were associated with increased consumption of processed food and sugar-sweetened beverages. Second, granting import licenses for domestically patented drugs was correlated with increased access to pharmaceuticals. Third, implementing trade agreements and associated policies was correlated with higher cardiovascular disease incidence and higher BMIs, whilst correlations with under-five mortality, maternal mortality, tuberculosis, and life expectancy were inconclusive. Fourth, the methodological quality of the studies we reviewed was predominately ‘weak’ or ‘moderate’ (11 out of 17 studies). Finally, there was weak co-citation between studies from public health with economics and psychology.

Our review has a number of limitations. Due to heterogeneity in measurement methods, research designs and outcome variables it was not possible to perform a meta-analysis or calculate pooled effect sizes. Further, we restricted our analysis to quantitative studies in order to evaluate the evidence of causal effects, yet qualitative studies can also provide useful insights into the mechanisms through which RTAs might affect peoples’ health.

We identified multiple limitations to existing studies. First, the majority of studies that examined the consequences of RTAs on food, beverage and tobacco consumption and access to pharmaceuticals were descriptive bivariate analyses. There is possible omitted variable bias, since trade agreements are often implemented as a consequence of, or alongside, other reforms [51] and macro-economic changes [52, 53]. Second, studies with stronger methodological designs tended to analyse the effects of trade agreements and policies using measures with weak specificity. The economic globalisation component of KOF index, used in four studies, can vary as a consequence of changes to both flows and restrictions on trade and investment [50]. A second approach, constructing a dichotomous indicator, treats RTAs as a ‘black-box’: it is unclear which policies within RTAs account for the outcome in question.

Third, the mechanisms that mediate links between RTAs and health were seldom explored. Fourth, there was a strong reliance on country-level data, limiting a full understanding of the social groups in whom the health effects of RTAs are concentrated. Fifth, there are possible health impacts of RTAs via myriad yet unexplored pathways that are identified in our conceptual framework. This includes benefits and harms to health due to changes in alcohol consumption, employment security, regulation, and health-services [13, 14, 54–56]. Sixth, our co-citation analysis identified opportunities for greater inter-disciplinary collaboration.

## Conclusions

These limitations notwithstanding, the systematic review identifies a common association between implementing RTAs or related trade and investment policies and higher consumption of processed foods and sugar-sweetened beverages, higher prevalence of cardiovascular diseases, and higher BMIs. Yet, considerable limitations in existing studies preclude definitive conclusions of causality. There is an opportunity for researchers to help advance public health practice and policy making worldwide by addressing potential endogeneity, analysing a broader range of mechanisms and outcomes, and identifying the specific policies within RTAs that affect people’s health.

## Additional file

Additional file 1: Supplemental information. (DOCX 55 kb)
