# Supplementary material for: The health impact of trade and investment agreements: a quantitative systematic review and network co-citation analysis
Source: Global Health. 2017 Mar 8;13:13. doi: 10.1186/s12992-017-0240-x (PMC5343316; doi:10.1186/s12992-017-0240-x)
Supplement: Additional file 1: — Supplemental information. (DOCX 55 kb) [file 12992_2017_240_MOESM1_ESM.docx]

**Web Appendix**

Appendix 1. PRISMA Checklist

Appendix 2. Quality appraisal tool

Appendix 3. Systematic review search operators

Appendix 4. Origin of published empirical studies of RTAs, trade policy and public health

Appendix 5. Network co-citation analysis for studies with minimum 5 cites

**Appendix 1. PRISMA Checklist**

| **Section/topic** | **#** | **Checklist item** | **Reported from page #** |
| --- | --- | --- | --- |
| **TITLE** | | | |
| Title | 1 | Identify the report as a systematic review, meta-analysis, or both. | 1 |
| **ABSTRACT** | | | |
| Structured summary | 2 | Provide a structured summary including, as applicable: background; objectives; data sources; study eligibility criteria, participants, and interventions; study appraisal and synthesis methods; results; limitations; conclusions and implications of key findings; systematic review registration number. | 2 |
| **INTRODUCTION** | | | |
| Rationale | 3 | Describe the rationale for the review in the context of what is already known. | 3-4 |
| Objectives | 4 | Provide an explicit statement of questions being addressed with reference to participants, interventions, comparisons, outcomes, and study design (PICOS). | 4-6 |
| **METHODS** | | | |
| Protocol and registration | 5 | Indicate if a review protocol exists, if and where it can be accessed (e.g., Web address), and, if available, provide registration information including registration number. | n/a |
| Eligibility criteria | 6 | Specify study characteristics (e.g., PICOS, length of follow-up) and report characteristics (e.g., years considered, language, publication status) used as criteria for eligibility, giving rationale. | 4-6 |
| Information sources | 7 | Describe all information sources (e.g., databases with dates of coverage, contact with study authors to identify additional studies) in the search and date last searched. | 4-6 |
| Search | 8 | Present full electronic search strategy for at least one database, including any limits used, such that it could be repeated. | 4-6 & 29 |
| Study selection | 9 | State the process for selecting studies (i.e., screening, eligibility, included in systematic review, and, if applicable, included in the meta-analysis). | 4-6 |
| Data collection process | 10 | Describe method of data extraction from reports (e.g., piloted forms, independently, in duplicate) and any processes for obtaining and confirming data from investigators. | 4-6 |
| Data items | 11 | List and define all variables for which data were sought (e.g., PICOS, funding sources) and any assumptions and simplifications made. | 4-6 |
| Risk of bias in individual studies | 12 | Describe methods used for assessing risk of bias of individual studies (including specification of whether this was done at the study or outcome level), and how this information is to be used in any data synthesis. | 4-6 |
| Summary measures | 13 | State the principal summary measures (e.g., risk ratio, difference in means). | n/a |
| Synthesis of results | 14 | Describe the methods of handling data and combining results of studies, if done, including measures of consistency (e.g., I^2^) for each meta-analysis. | 4-6 |
| Risk of bias across studies | 15 | Specify any assessment of risk of bias that may affect the cumulative evidence (e.g., publication bias, selective reporting within studies). | 12-14 |
| Additional analyses | 16 | Describe methods of additional analyses (e.g., sensitivity or subgroup analyses, meta-regression), if done, indicating which were pre-specified. | 4-6 |
| **RESULTS** | | | |
| Study selection | 17 | Give numbers of studies screened, assessed for eligibility, and included in the review, with reasons for exclusions at each stage, ideally with a flow diagram. | 4-6 and figure 3 |
| Study characteristics | 18 | For each study, present characteristics for which data were extracted (e.g., study size, PICOS, follow-up period) and provide the citations. | 6-10 |
| Risk of bias within studies | 19 | Present data on risk of bias of each study and, if available, any outcome level assessment (see item 12). | 6-12 |
| Results of individual studies | 20 | For all outcomes considered (benefits or harms), present, for each study: (a) simple summary data for each intervention group (b) effect estimates and confidence intervals, ideally with a forest plot. | 6-10 |
| Synthesis of results | 21 | Present results of each meta-analysis done, including confidence intervals and measures of consistency. | n/a |
| Risk of bias across studies | 22 | Present results of any assessment of risk of bias across studies (see Item 15). | 11-12 |
| Additional analysis | 23 | Give results of additional analyses, if done (e.g., sensitivity or subgroup analyses, meta-regression [see Item 16]). | 10-12 |
| **DISCUSSION** | | | |
| Summary of evidence | 24 | Summarize the main findings including the strength of evidence for each main outcome; consider their relevance to key groups (e.g., healthcare providers, users, and policy makers). | 12-14 |
| Limitations | 25 | Discuss limitations at study and outcome level (e.g., risk of bias), and at review-level (e.g., incomplete retrieval of identified research, reporting bias). | 12-14 |
| Conclusions | 26 | Provide a general interpretation of the results in the context of other evidence, and implications for future research. | 12-14 |
| **FUNDING** | | | |
| Funding | 27 | Describe sources of funding for the systematic review and other support (e.g., supply of data); role of funders for the systematic review. | n/a |

*Source:*  Moher D, Liberati A, Tetzlaff J, Altman DG, The PRISMA Group (2009). Preferred Reporting Items for Systematic Reviews and Meta-Analyses: The PRISMA Statement. PLoS Med 6(6).

**Appendix 2. Quality Appraisal Tool**

**STUDY DESIGN**

(**Q1**) The study design is:

1. Experimental or quasi-experimental
   - 1. Individual-randomised
     2. Group-randomised
     3. Non-randomised
2. Observational
   - 1. Cross-sectional
     2. Longitudinal
     3. Case-control
     4. Quasi- or natural-experiment
3. Any other method or did not state method (i.e. pre-post test without control group)

(**Q2**) Was this an intervention study?

**Yes** – proceed

**No** – go to question 7

(**Q3**) Is the intervention of interest clearly described?

1. Yes
2. No

(**Q4**) Were (groups of) subjects randomized into intervention groups?

1. Yes
2. No
3. Not applicable

(**Q5**) Was the intervention assignment concealed from participants and care givers until recruitment was completed?

1. Yes
2. No
3. Can’t tell

(**Q6**) Was (were) the intervention or exposure status of participants concealed from the outcome assessors?

1. Yes
2. No
3. Can’t tell

(**Q7**) Were power/sample size calculations conducted?

1. Yes, details of calculation provided
2. Yes, no details provided
3. Not reported or post hoc computation
4. Not applicable (using an existing database and referring to design article*

**Rating study design: Strong:** Q1 is 1

**Moderate:** Q1 is 2

**Weak:** Q1 is 3

**Rating blinding: Strong:** Q5 and Q6 are 1

**Moderate:** Q5 or Q6 is 1**; or** Q5 or Q6 are 3

**Weak:** Q5 and Q6 are 2; **or** Q5 and Q6 are 3

**(no rate is given when study is not an intervention study)**

*• If the study is using data from a large existing database such as HSE, NHANES, BRFSS etc, often the authors refer to the design paper of the original study and no information in the present article is being described about power calculations, validity of tools et.*

**REPRESENTATIVENESS (selection bias)**

(**Q8**) Is the spectrum of individuals selected to participate likely to be representative of the wider population who experience the intervention/exposure/situation?

1. Very likely
2. Somewhat likely
3. Not likely (selected group of users e.g., volunteers)
4. Can´t tell (no information provided)
5. Not applicable (using an existing database and authors refer to design article)

(**Q9**) What percentage of the selected participants agreed to participate?

1. ⁮ …………..%
2. ⁮ Can’t tell
3. ⁮ Not applicable

(**Q10**) Were inclusion/exclusion criteria specified and number of exclusions reported?

1. Criteria and number of exclusions reported
2. Criteria or number of exclusions not reported
3. Criteria and number not reported

**Rating: Strong:** Q8 is 1

**Moderate:** Q8 is 2

**Weak:** Q8 is 3 or 4

**No rating:** Q8 is 5

**REPRESENTATIVENESS (withdrawals and drop-outs)**

(**Q11**) Were withdrawals and drop-outs reported in terms of numbers and reasons per group?

1. Numbers and reasons provided
2. Numbers but no reasons provided
3. Can’t tell (if longitudinal data)
4. Not applicable (if cross-sectional data or if using an existing database and authors refer to design article)

*If Q11 is 1 or 2, proceed to Q12. Otherwise, proceed to Q13.*

(**Q12**) What was the loss to follow-up/percentage completing the study? (If % differs by groups, record the lowest)

1. ⁮ …………..%
2. ⁮ Not provided
3. ⁮ Not applicable

**Rating: Strong:** Q11 is 1

**Moderate:** Q11 is 2

**Weak:** Q11 is 3

**No rating:** Q11 is 4

**CONFOUNDERS**

(**Q13#**) What confounders were the analyses adjusted for?

…………………………………………………………………………………………………...

…………………………………………………………………………………………………...

(**Q13**) Were analyses appropriately adjusted for confounders?

1. For most confounders
2. For some confounders
3. No or can’t tell

*The following are examples of confounders: race, sex, marital status/family, age, SES (income or class), education, health status, pre-intervention score on outcome measure.*

*Considering the study design, were appropriate methods for controlling confounding variables and limiting potential biases used? Confounding can be addressed by appropriate use of randomization, restriction, matching, stratification, or multivariable methods. Sometimes use of a single method may be inadequate. Some biases can be limited by institution of data collection or study procedures that support validity of the study (e.g. training and/or blinding of interviewers or observers, interviewers and observers are different from interventions’ implementers etc). Example: if between-group differences persist after randomization or matching, statistical control should also have been used.*

**Rating: Strong:** Q13 is 1

**Moderate:** Q13 is 2

**Weak:** Q13 is 3

**DATA COLLECTION**

(**Q14**) Were validity, reliability or appropriateness of the data collection tools discussed?

- 1. Both validity and reliability were discussed
  2. a. Validity or reliability were discussed

b. A national dataset was used and authors provided adequate information to find information on validity and reliability

- 1. None of them were discussed

**Rating: Strong:** Q14 is 1

**Moderate:** Q14 is 2

**Weak:** Q14 is 3

**DATA ANALYSIS**

(**Q15**) Were appropriate statistical analyses conducted (including correction for multiple tests where applicable)?

1. a. Statistical methods were described and were appropriate and comprehensive – sophisticated approach

b. Statistical methods were described and were appropriate and comprehensive –simple approach

1. Statistical methods were described and less appropriate
2. No description of statistical methods or inappropriate methods

**Rating: Strong:** Q15 is 1

**Moderate:** Q15 is 2

**Weak:** Q15 is 3

**REPORTING**

(**Q16**) Are the hypothesis/aim/objective of the study clearly described?

1. Yes
2. No

(**Q17**) Are the main outcomes to be measured clearly described?

1. Yes
2. No

(**Q18**) Are the main findings clearly described?

1. Yes
2. No

(**Q19**) Have actual probability values been reported

*(i.e. p=0.345 instead of p>0.05; same goes for t-values, 95%CIs etc)?*

1. Yes
2. No

**Rating: Strong:** Q16 and Q19 are 1

**Moderate:** Q16 or Q19 are 1

**Weak:** Q16 and Q19 are 2

Studies have six component ratings. The overall rating for each study is determined by assessing the component ratings.

**Strong** will be attributed to those with no WEAK ratings and at least three STRONG ratings; **Moderate** will be given to those with one WEAK rating or fewer than three STRONG ratings;

**Weak** will be attributed to those with two or more WEAK ratings.

The final decision of both reviewers will be: **strong, moderate, or weak**.

**Appendix 3. Systematic review search operators**

Boolean operators were used to combine the below search terms:

Search terms

trade liberalisation; liberalization

investment liberalisation; liberalization

trade agreements; agreement

investment agreements; agreement

bilateral; bi-lateral agreements; agreement

multilateral; multi-lateral; agreemts; agreement

preferential trade agreement; PTA

trade and investment agreement; TIA

regional trade agreement; RTA

trade policy; policies

investment policy; policies

globalisation

globalization

regionalism

multilateralism

multinationalism

economic integration

health

health service; health-service; health-services

healthcare; health-care

food

nutrition

alcohol

tobacco

social policy

social protection

health policy

disease

chronic disease

chronic diseases

non-communicable disease

non communicable disease

NCD

NCDs

**Appendix 4. Origin of published empirical studies of RTAs, trade policy and public health**

| **Journal, book or report title** | **Number of studies** | **Category** |
| --- | --- | --- |
| Globalization and Health | 3 | Globalization and Health |
| Globalisation, agriculture and development: perspectives from asia-pacific | 1 | Book chapter |
| Trade, food, diet, and health : perspectives and policy options | 1 | Book chapter |
| NBER working paper 5543 | 1 | Empirical study for policy report |
| WHO Commission on Social Determinants of Health report | 1 | Empirical study for policy report |
| Critical Public Health | 1 | Public health |
| Health Policy and Planning | 1 | Public health |
| International Journal of Health Planning Management | 1 | Public health |
| PLoS Medicine | 1 | Public health |
| Progress in Cardiovascular Diseases | 1 | Public health |
| Public Health Nutrition | 1 | Public health |
| Tobacco Control | 1 | Public health |
| Pakistan Development Review | 1 | Social science |
| Social Science and Medicine | 1 | Social science |
| PLoS One | 1 | Multidisciplinary |
| **Total** | **17** |  |
